# Relationship Between Type of Delivery and Growth Trajectory in the First Year of Life: The Araraquara Cohort Study

**DOI:** 10.1002/ajhb.70075

**Published:** 2025-06-09

**Authors:** Roseanne de Sousa Nobre, Paula Louro Silva, Letícia Falcão de Carvalho, Jéssica Lana Sales Lacerda, Lívia Patrícia Rodrigues Batista, Tamiris Ramos‐Silva, Natália Pinheiro‐Castro, Liania Alves Luzia, Patrícia Helen de Carvalho Rondó

**Affiliations:** ^1^ Postgraduate Epidemiology Programme School of Public Health, University of São Paulo São Paulo Brazil; ^2^ Postgraduate Public Health Nutrition Programme School of Public Health, University of São Paulo São Paulo Brazil; ^3^ Postgraduate Public Health Programme School of Public Health, University of São Paulo São Paulo Brazil; ^4^ Nutrition Department School of Public Health, University of São Paulo São Paulo Brazil

**Keywords:** cesarean delivery, growth, growth trajectory, infant obesity, weight gain trajectory

## Abstract

**Objective:**

The relationship between cesarean delivery and infant growth is controversial. Therefore, the aim of this study was to evaluate the association between the type of delivery and the growth trajectory of Brazilian infants in the first year of life.

**Materials and Methods:**

This was a prospective cohort study conducted between 2016 and 2021 as part of the Araraquara Cohort Study that assessed quarterly anthropometry of 719 and 667 infants to evaluate, respectively, the effects of type of delivery on weight gain velocity (WGV) and length gain velocity (LGV) using generalized estimating equations.

**Results:**

The type of delivery was not associated with WGV or LGV in the first year of life. Higher mean WGV was observed among infants born to mothers with higher education, male infants, formula‐fed infants, and those with the lowest birth weight, while infants with diarrhea had lower mean WGV. Higher mean LGV was found among formula‐fed infants and infants born to taller mothers, while infants with the highest length at birth had lower LGV.

**Conclusion:**

The factors that explain WGV and LGV in this population are more closely related to maternal characteristics such as height and educational attainment, birth characteristics, feeding behavior, and morbidity than to the type of delivery.

## Graphical abstract

Graph 1 Type of delivery and weight gain velocity (WGV) in the first year of life. Exposure = Cesarean delivery. Outcome = WGV, Confounders = sex, birth weight, maternal BMI, maternal education and per capita income; Variables associated with the exposure or with the outcome = breastfeeding in the first hour, marital status, breastfeeding, hospitalization, diarrhea, respiratory infection and anemia. Type of delivery and length gain velocity (LGV) in the first year of life. Exposure = Cesarean delivery. Outcome = LGV, Confounders = sex, length at birth, maternal height, maternal education and per capita income); Variables associated with the exposure or with the outcome = breastfeeding in the first hour, marital status, breastfeeding, hospitalization, diarrhea, respiratory infection and anemia.

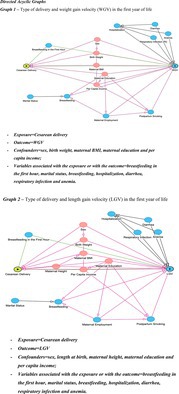

## 1 Introduction

Growth from fertilization to adulthood exhibits different growth velocities; some seem accelerated compared to others (e.g., growth in utero vs. growth in mid‐childhood).

Growth in childhood has been associated with factors, such as maternal nutritional and socioeconomic status (Koletzko et al. 2019; Ellsworth et al. 2020; Mumm et al. 2017; Addo et al. 2013; Karlsson and Dribe 2022; Nguyen et al. 2012), sex (Lampl and Jeanty 2003; Johnson et al. 2013), birth weight (Griffin et al. 2016), length at birth (Morris et al. 1998), breastfeeding (Ellsworth et al. 2020), morbidity (Ofek et al. 2022), hospitalization (Richard et al. 2013) and postpartum smoking (Banderali et al. 2015).

The first year of life is the phase characterized by rapid growth, when individuals are most susceptible to nutritional problems. Infants usually triple their birth weight and grow 50% in length by 1 year, signaling healthy organ and skeletal development (Tanner and Preece 1989). Monitoring growth during this period (Regnault et al. 2010) is extremely important to identify and correct deficits early in order to minimize their harmful and long‐lasting impact (Suzuki 2018). Therefore, many health organizations, including the World Health Organization (WHO), routinely recommend monitoring growth (WHO 2009). If possible, infants should be assessed monthly during the first year of life and less frequently thereafter. In addition to the usual indicators for assessing nutritional status such as weight, length, and body mass index (BMI), growth velocity can also be used. This indicator is calculated by weight and length over a specific period of time and has numerous advantages compared to weight and length assessed at different times in the post‐natal period: (a) it is more sensitive to growth abnormalities and nutritional deficits; (b) it reflects more precisely the growth patterns; (c) it predicts future problems such as short stature; (d) it allows comparison between different populations, considering that variations in growth can be due to genetic, environmental, or nutritional issues; (e) it is important for decision‐making among clinicians (Dewey and Begum 2011; Olusanya and Renner 2011; Bozzola and Meazza 2012).

Some authors have suggested cesarean section to be linked to growth in infancy (Mueller et al. 2019; Rolfs et al. 2017). This is an important topic for research, considering that cesarean rates are increasing worldwide, particularly in low‐ and middle‐income countries (Boerma et al. 2018; Martin et al. 2019), regions where the impact of this procedure can be more concerning for mothers and their children. Infants born by cesarean section showed higher weight and length gain in the first year of life than those born by vaginal delivery (Mueller et al. 2019). The accelerated growth of children born by cesarean section may be explained by the programming of immunological and metabolic factors early in life by the intestinal microbiota (Mueller et al. 2017; Selma‐Royo et al. 2020). In addition to potentially being associated with early alterations in the biodiversity of gut bacteria (Biasucci et al. 2008), Yoshimitsu et al. (2000) observed higher concentrations of leptin in the umbilical cord of newborns delivered by cesarean section compared to those delivered vaginally. However, the findings of studies on the effect of type of delivery on growth in the first years of life are controversial (Vieira et al. 2015; Rolfs et al. 2017; Mueller et al. 2017; Kelly et al. 2020; Babu et al. 2021; Martin et al. 2019). Notably, most of the studies that found no association between type of delivery and anthropometric measurements in infancy did not consider the velocity of weight or length gain but rather associations at different time points (Masukume et al. 2019; Dal'Maso et al. 2022).

Brazil has one of the world's highest cesarean section rates (Boerma et al. 2018), with Ministry of Health data indicating that approximately 57% of births occur via C‐section (Ministério da Saúde 2022). This trend is driven by cultural preferences, perceived safety benefits, and logistical conveniences (Muhandule et al. 2024; Velho et al. 2019; Lansky et al. 2019). As a middle‐income country and one of the few nations where cesarean deliveries outnumber vaginal births (Betrán et al. 2021), Brazil offers a critical context to study how delivery method relates to infant growth trajectories during the first year of life. Such research is urgently needed to address global gaps in understanding child growth patterns, which vary significantly across populations (Veile et al. 2019).

## 2 Materials and Methods

This is a prospective cohort study that is part of the Araraquara Cohort Study (Victor et al. 2024), which followed up infants from birth to 1 year of life in Araraquara city, São Paulo State, Brazil. Those infants were the offspring of women who were selected at the beginning of their pregnancy from 42 Health Units in Araraquara and the surrounding region. They were covered by the National Health Service (SUS), which assists low‐income families, and comprise a representative sample of the low‐ to middle‐income population of the municipality.

All infants from the Araraquara Cohort Study, born between 2017 and 2021, at a public reference maternity hospital for Araraquara and the surrounding region were included in the study. The majority of the births in the city take place at this maternity. Demographic, socioeconomic, obstetric, and nutritional data were collected from the mothers in the third trimester of pregnancy. Infants with congenital diseases that compromised feeding after birth, infants of multiple pregnancies, preterm infants (< 37 weeks), and infants with proven cognitive impairment were excluded.

Information about the type of delivery, maternal and infant conditions during delivery and the postpartum period, as well as infant birth weight and length were collected by the researchers from the maternity records. Measurements of the infants were taken between 12 and 72 h postpartum and at 3, 6, 9, and 12 months of life by three trained researchers at the Araraquara Special Health Service (SESA), School of Public Health, University of São Paulo. While still in the maternity ward and shortly before the infant's appointments at SESA, the mothers were asked about breastfeeding habits, and infant morbidity, especially anemia, diarrhea, and respiratory infections, using questionnaires administered after the anthropometric measurements of the infants. At the maternity and the 42 Health Units women were encouraged to breastfeed their children, supported by the *Breastfeed and Feed Brazil Strategy* (*Estratégia Amamenta e Alimenta Brasil*), a national program implemented in 2012 by the Brazilian Ministry of Health (Ministério da Saúde 2012). Data regarding morbidity were checked, when necessary, by requesting the names of the medications prescribed by the infant's physicians.

Based on weight, gestational age, and sex, newborns were classified as small for gestational age (SGA), adequate for gestational age (AGA), and large for gestational age (LGA) when weight was below the 10th percentile, between the 10th and 90th percentile, and above the 90th percentile, respectively, according to the Intergrowth reference curves (Villar et al. 2015). Infant weight and length were measured using a Soehnle Multina Plus digital scale (Soehnle, Hamburg, Germany) and a Seca 416 infantometer (Seca, Hamburg, Germany), respectively, following Jelliffe and Jelliffe (1989) guidance. All measurements were performed in duplicate according to the recommendations of Norris (2022) and Jelliffe (1989).

To assess inter and intra observer variations, a sample of 20 infants was measured four times by all three researchers, in 10 min intervals, and all the results were within 5% in all repeated measurements.

The weight gain velocity (WGV) and length gain velocity (LGV) of the infants were assessed from birth to the third (T1), sixth (T2), ninth (T3), and twelfth (T4) months of life using the following formulas:

WGV (g/day) = (weight at T1, T2, T3 and T4 – birth weight)/age in days;

LGV (cm/month) = (length at T1, T2, T3 and T4 – birth length)/age in days (Nguyen et al. 2012).

Statistical analyses were performed using the SPSS 20.0 statistical program. For descriptive analysis, explanatory variables were presented as frequencies and measures of central tendency (mean) and variability (standard deviation). The Kolmogorov–Smirnov test was used to test for normality of the outcome variables (WGV and LGV). Differences between categorical and continuous variables were analyzed using the Chi‐square test and Student's *t*‐test, respectively. Generalized estimating equations (GEE) were applied to evaluate the effect of time and type of delivery on the WGV and LGV of infants at different time points between 0 and 1 year of age, using two analyses for the outcome variables: WGV and LGV. GEE is a statistical approach that takes into account the correlation between repeated measurements of the same individual over time and is suitable for this study. Unlike traditional regression models, GEE adjusts several models for longitudinal data, considering the individual as a random effect. This means that individual variations are incorporated into the model as part of the correlation structure, allowing a robust analysis even in situations where the data do not follow a normal distribution (Gardiner et al. 2009; Guimarães Santos Pinto and Naomi Hirakata 2012).

Directed acyclic graphs—DAG (Greenland et al. 1999; Rohrer 2018) were created to better explain the relationship between the exposure variable (type of delivery), the outcomes (WGV and LGV) and covariates (confounding variables and those variables associated only with the exposure variable or with the outcomes) (DAG1, DAG2). In both GEE analyses (considering the outcomes WGV and LGV), all covariates (or independent variables) listed in the Directed Acyclic Graphs (DAGs) were initially included, and some of them were subsequently removed using the backward method (Hosmer et al. 2013). The variables with statistically significant associations with the outcome variables (*p* < 0.05) or proven biological relevance were maintained in the final models.

To assess the average effect of type of delivery on WGV and LGV at T1, T2, T3, and T4, vaginal delivery was set as the reference standard. The Bonferroni correction was used to adjust *p*‐values for the multiple comparisons. A level of significance of 5% was adopted in all statistical analyses.

## 3 Results

**FIGURE 1:**
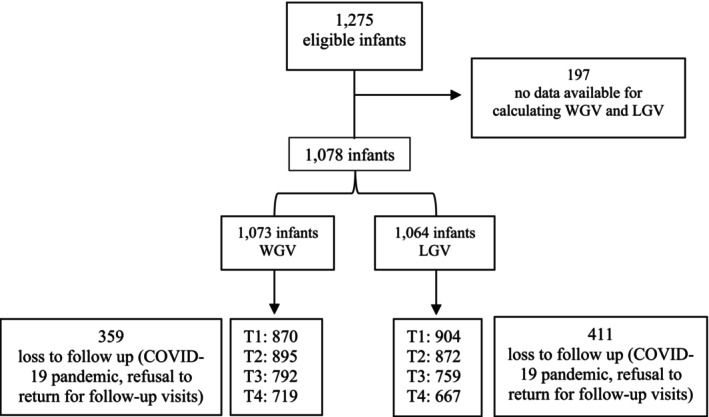
Follow‐up of the study participants. WGV = weight gain velocity; LGV = length gain velocity; T1 = 3 months; T2 = 6 months; T3 = 9 months; T4 = 12 months.

A total of 1275 infants met the eligibility criteria; however, data at two critical time points required for the calculation of WGV and LGV were missing for 197 (15.45%) infants. Thus, 1078 infants were included in the final sample; data of at least two time points necessary for the calculation of WGV and LGV were available for 1073 and 1064 infants, respectively. At 1 year of age, there were 359 (28.16%) and 411 (32.24%) losses to follow up of infants with WGV and LGV data, respectively. The distribution of participants with available data at the four specific time points is presented in Figure 1.

Table 1 shows the comparison of demographic, socioeconomic, and nutritional characteristics between the population studied and participants lost to follow‐up in the first year of life. No differences in these characteristics were observed between populations.

**TABLE 1:** Demographic, socioeconomic, and nutritional characteristics of the population studied and participants lost to follow‐up.

	Weight			Length		
	Population studied (*n* = 719)	Lost to follow‐up (*n* = 359)	*P*	Population studied (*n* = 667)	Lost to follow‐up (*n* = 411)	*p*
Maternal characteristics						
Age (years)	27.63 (6.44)	27.15 (6.56)	0.256	27.68 (6.47)	27.14 (6.51)	0.182
≤ 19	92 (12.8)	50 (13.9)		85 (12.7)	57 (13.9)	
20–34	519 (72.2)	260 (72.4)		479 (71.8)	300 (73.0)	
≥ 35	108 (15.0)	49 (13.7)		103 (15.5)	54 (13.1)	
Ethnicity			0.264			0.324
White	321 (44.6)	177 (49.3)		304 (45.6)	195 (47.4)	
Non‐white*	398 (55.4)	182 (50.7)		363 (54.4)	216 (52.6)	
Marital status			0.505			0.771
Married/stable union	650 (90.4)	315 (87.7)		602 (90.3)	363 (88.3)	
Single/separated/widowed	69 (9.6)	44 (12.3)		65 (9.7)	48 (11.7)	
Education (years)			0.488			0.163
≤ 4	10 (1.4)	3 (0.8)		9 (1.3)	4 (1.0)	
5–8	82 (11.4)	56 (15.6)		70 (10.5)	68 (16.5)	
9–11	510 (70.9)	237 (66.0)		476 (71.4)	273 (66.4)	
≥ 12	117 (16.3)	63 (17.6)		112 (16.8)	66 (16.1)	
Monthly per capita income (R$)**	747.70 (501.04)	728.49 (462.52)	0.543	761.46 (508.08)	706.98 (663.24)	0.075
Newborn characteristics						
Weight (g)	3268.45 (425.29)	3286.73*** (453.69)	0.519	3273.01 (423.56)	3276.96*** (452.97)	0.885
Length (cm)	49.14 (2.79)	49.27*** (2.14)	0.338	49.21 (2.10)	49.15*** (2.14)	0.670

*Note:* Data are presented as mean (standard deviation, SD) or frequencies. *Non‐white: Black, Asian, Hispanic, mixed race. **R$1.00 = US$4.41. ***n = 7 missing.

Table 2 shows the maternal and newborn characteristics. Most infants were born vaginally, whether they were followed up for WGV (52.4%) or LGV (52.3%). Maternal age (*p* < 0.001), BMI in the third trimester of pregnancy (*p* < 0.001), Height (*p* < 0.024), AGA (*p* = 0.003) and LGA (*p* < 0.001) showed statistically significant differences between cesarean delivery and vaginal delivery.

**TABLE 2:** Maternal and infant characteristics according to type of delivery.

	Weight (*n* = 719)			Length (*n* = 667)		
	Vaginal delivery (*n* = 377)	Cesarean section (*n* = 342)	*p*	Vaginal delivery (*n* = 349)	Cesarean section (*n* = 318)	*p*
Maternal characteristics						
Age (years)	26.53 (6.33)	28.86 (6.35)	≤ 0.001	26.67 (6.35)	28.86 (6.41)	≤ 0.001
≤ 19	60 (15.9)	32 (9.4)		40 (11.5)	31 (9.7)	
20–34	273 (72.4)	246 (71.9)		267 (76.5)	226 (71.1)	
≥ 35	44 (11.7)	64 (18.7)		42 (12.0)	61 (19.2)	
Ethnicity			0.797			0.435
White	164 (43.5)	157 (45.9)		152 (43.6)	152 (47.8)	
Non‐white*	213 (56.5)	185 (54.1)		197 (56.4)	166 (52.2)	
Marital status			0.517			0.53
Married/stable union	346 (91.8)	304 (88.9)		321 (92.0)	281 (88.4)	
Single/separated/widowed	31 (8.2)	38 (11.1)		28 (8.0)	37 (11.6)	
Education (years)			0.279			0.873
≤ 4	6 (1.6)	4 (1.2)		5 (1.42)	4 (1.3)	
5–8	42 (11.1)	40 (11.7)		33 (9.5)	37 (11.6)	
9–11	268 (71.1)	243 (71.0)		251 (71.9)	224 (70.4)	
≥ 12	61 (16.2)	55 (16.1)		60 (17.2)	53 (16.7)	
Monthly per capita income (R$)**	753.96 (496.54)	740.81 (506.58)	0.675	777.61 (500.41)	740.99 (517.87)	0.355
Height (cm)	162.40 (5.83)	161.12 (6.34)	0.005	162.43 (5.81)	161.36 (6.42)	0.024
BMI (kg/m^2^)	27.97 (4.82)	30.60 (5.40)	≤ 0.001	27.99 (4.77)	30.61 (5.33)	≤ 0.001
Underweight	5 (1.3)	1 (0.3)		3 (0.9)	1 (0.3)	
Normal weight	110 (29.2)	54 (15.8)		104 (29.8)	48 (15.1)	
Overweight	162 (43.0)	127 (37.1)		150 (43)	119 (37.4)	
Obese	100 (26.5)	160 (46.8)		92 (26.4)	150 (47.2)	
Employment			0.732			0.598
Yes	128	112		122 (35.0)	105 (33.0)	
No	249	230		227 (65.0)	213 (67.0)	
Postpartum smoking			0.680			0.793
Yes	19	15		18 (5.2)	15 (4.7)	
No	358	327		331 (94.8)	303 (95.3)	
Newborn characteristics						
Sex			0.929			0.733
Female	190 (50.4)	173 (50.6)		173 (49.6)	164 (51.6)	
Male	187 (49.6)	169 (49.4)		176 (50.4)	154 (48.4)	
Apgar (5th minute)	8.59 ± 1.19	8.50 (1.28)	0.445	8.57 (1.24)	8.52 (1.29)	0.617
GA (weeks)	39.59 ± 1.02	39.45 (1.14)	0.191	39.58 (1.03)	39.48 (1.15)	0.359
Weight (g)	3243.48 (375.55)	3296.32 (474.38)	0.097	3243.68 (369.57)	3306.16 (475.74)	0.059
Length (cm)	49.21 (2.01)	49.07 (3.47)	0.368	49.28 (1.99)	49.13 (2.22)	0.336
Weight classification by GA***						
SGA	33 (8.8)	40 (11.7)	0.179.	31 (8.9)	36 (11.3)	0.277
AGA	321 (85.1)	266 (77.8)	0.001	298 (85.4)	249 (78.3)	0.003
LGA	23 (6.1)	36 (10.5)	0.028	20 (5.7)	33 (10.4)	0.039
Anemia			0.642			0.527
Yes	16 (4.2)	17 (5.0)		15 (4.3)	17 (5.3)	
No	361 (95.8)	325 (95.0)		334 (95.7)	301 (94.7)	
Respiratory infections			0.482			0.657
Yes	7 (1.9)	9 (2.6)		7 (2.0)	8 (2.5)	
No	370 (98.1)	333 (97.4)		342 (98.0)	310 (97.5)	
Diarrhea			0.157			0.096
Yes	82 (21.8)	60 (17.5)		82 (23.5)	58 (18.2)	
No	295 (78.2)	282 (82.5)		267 (76.5)	260 (81.8)	
Hospitalization			0.453			0.449
Yes	7 (1.9)	4 (1.2)		7 (2.0)	4 (1.3)	
No	370 (98.1)	338 (98.8)		342 (98.0)	314 (98.7)	

*Note:* Data are presented as mean (standard deviation, SD) or frequencies. *Non‐white: Black, Asian, Hispanic, mixed race. **R$1.00 = US$4.41. ***[18].

Abbreviations: AGA = adequate for gestational age; BMI = body mass index (evaluated in the third trimester); GA = gestational age; LGA = large for gestational age; SGA = small for gestational age.

As can be seen in Table 3, type of delivery was not associated with WGV or LGV in the first year of life.

**TABLE 3:** Weight gain velocity (WGV) and length gain velocity (LGV) of infants at 3, 6, 9, and 12 months of age according to type of delivery.

	Cesarean delivery				Vaginal delivery				
	T1	T2	T3	T4	T1	T2	T3	T4	*P*
WGV (g/day)	29.84 (29.12; 30.56)	23.65 (23.18; 24.12)	19.88 (19.47; 20.28)	17.03 (16.67; 17.40)	29.94 (29.24; 30.64)	24.12 (23.64; 24.59)	19.89 (19.53; 20.25)	16.93 (16.61; 17.25)	0.672
LGV (cm/month)	3.64 (3.62; 3.78)	2.95 (2.91; 2.99)	2.42 (2.39; 2.46)	2.13 (2.10; 2.16)	3.64 (3.56; 3.71)	2.96 (2.91; 3.01)	2.39 (2.36; 2.43)	2.12 (2.09; 2.15)	0.378

*Note:* Data are presented as mean (confidence interval). T1 = 3 months; T2 = 6 months; T3 = 9 months; T4 = 12 months.

Table 4 shows that maternal education (*p* < 0.001), sex (*p* < 0.001), birth weight (p < 0.001), formula feeding (*p* = 0.004), and diarrhea (*p* = 0.041) were associated with WGV. The beta coefficients represent differences in means or slopes relative to the reference value (intercept). WGV was inversely associated with the time of assessment, being lower at T2 (−5.9 g/day), T3 (−10 g/day), and T4 (−12.9 g/day) compared to the reference T1, and inversely associated with birth weight (−0.001 g/day) and diarrhea (−0.57 g/day). Boys had a WGV 2.19 g/day higher than girls, and infants of women with 5 to 8 and 9 to 11 years of education showed higher WGV of 1.1 and 1.2 g/day, respectively.

**TABLE 4:** Generalized estimating equations to evaluate the effect of vaginal and cesarean deliveries on weight gain velocity at T1, T2, T3, and T4.

Model	Beta (β)^1^	95% CI	Wald test	*P*
T1 (reference)				
T2	−5.996	−6.441; −5.551	697.810	< 0.001
T3	−10.044	−10.522; −9.565	1693.004	< 0.001
T4	−12.902	−13.434; 12.369	2254.026	< 0.001
Vaginal delivery (reference)				
Cesarean section	−0.194	−0.751; 0.363	0.467	0.494
≥ 12 years of education (reference)				
9–11	1.156	0.471; 1.842	10.928	< 0.001
5–8	1.227	0.213; 2.242	5.619	0.018
≤ 4	1.973	−1.789; 4.262	0.641	0.423
Female (reference)				
Male	2.199	1.635; 2.763	58.373	< 0.001
Birth weight (g)	−0.001	−0.002; −0.001	15.478	< 0.001
Breastfed (reference)				
Formula fed	0.820	0.269; 1.370	8.516	0.004
Without diarrhea (reference)				
Diarrhea	−0.572	−1.122; −0.023	4.166	0.041
Intercept	34.188	30.600; 37.776	348.822	< 0.001

*Note:* Final adjusted model. T1 = 3 months; T2 = 6 months; T3 = 9 months; T4 = 12 months. CI = confidence interval.

LGV was affected by maternal height (*p* < 0.001), length at birth (*p* < 0.001), and formula feeding (*p* < 0.001) (Table 5). LGV was inversely associated with the time of assessment, being lower at T2 (−0.70 cm/month), T3 (−1.24 cm/month), and T4 (−1.53 cm/month) compared to the reference T1, and inversely associated with length at birth (−0.114 cm/month). Maternal height was positively associated with the infant's LGV. Each additional centimeter in maternal height was associated with a small but significant increase in infant length (0.009 cm/month). Formula‐fed infants had an LGV 0.08 cm/month higher than breastfed infants.

**TABLE 5:** Generalized estimating equations to evaluate the effect of vaginal and cesarean deliveries on length gain velocity at T1, T2, T3, and T4.

Model	Beta (β)1	95% CI	Wald test	*p*
T1 (reference)				
T2	−0.701	−0.751; −0.651	753.326	< 0.001
T3	−1.239	−1.291; −1.187	2.159.808	< 0.001
T4	−1.533	−1.591; −1.476	2.697.840	< 0.001
Vaginal delivery (reference)				
Cesarean section	−0.001	−0.052; 0.050	0.002	0.965
Maternal height (cm)	0.009	0.005; 0.013	18.796	< 0.001
Length at birth (cm)	−0.114	−0.127; −0.100	273.832	< 0.001
Breastfed (reference)				
Formula fed	0.088	0.038; 0.137	12.164	< 0.001
Intercept	7.858	6.953; 8.764	289.294	< 0.001

*Note:* Final adjusted model. T1 = 3 months; T2 = 6 months; T3 = 9 months; T4 = 12 months. CI = confidence interval.

## 4 Discussion

Type of delivery was neither associated with WGV nor with LGV. In agreement with our findings, three studies also conducted in Brazil, two of them prospective cohorts (De Lima Lins and Pedraza 2021; Rolfs et al. 2017) involving respectively 144 and 240 infants, also found no association between type of delivery and WGV or LGV in children followed up over the first 6 months of life. In a cross‐sectional study with 371 infants, Vieira et al. (2015) also found no association of weight or length velocities with type of delivery, even after controlling for maternal and infant variables. It should be noted that the results of these studies are limited since they only assessed growth for up to 6 months of life, and only Vieira et al. (2015) used robust statistical methods to control for confounding factors.

In contrast, other studies have shown that cesarean section is a risk factor for greater weight and length in the first year of life (Mueller et al. 2019). This finding may be partly explained by disruption of the intestinal microbiota, which alters early life metabolism and accelerates growth in the infant born by cesarean section (Mueller et al. 2019; Li et al. 2013; Kuhle et al. 2015). Studies have also attributed growth differences between infants born by normal delivery and cesarean section to the impact of the latter on stress hormones and cytokine concentrations (Mueller et al. 2019). However, further longitudinal studies are needed to confirm these hypotheses (Mueller et al. 2019). The prevalence of overweight and obesity among 1‐year‐old children included in our study was approximately 12.3% and 11.9%, respectively, although there was no significant difference between those born via cesarean delivery and vaginal delivery (data not shown).

The lack of a relationship between type of delivery and growth in the first year of life observed in the present study might be due to differences in the indication for cesarean section in each case. In Brazil, elective or pre‐scheduled cesarean sections are frequent and, consequently, women do not necessarily go into labor to deliver their infants. It raises the question as to whether the infant is genuinely prepared for birth or if it requires additional weeks of growing inside the womb. The public maternity hospital in Araraquara, where the data collection took place, supports vaginal delivery and has a cesarean section rate of 44%, below the Brazilian average of 57%, but still considered high. The majority (84%) of cesarean sections performed at the maternity hospital were elective and planned (data not shown), with only 16% considered emergency. This population did not have access to a private health insurance; otherwise, the cesarean section rate would be much higher (Braga et al. 2023). There was a difficulty in differentiating between elective and planned cesarean sections, considering that the indications for these two types of cesarean sometimes overlapped, possibly due to the fact that cesareans in Brazil have become part of the obstetric routine.

Regarding maternal factors associated with the type of delivery, we observed that the higher the maternal education, the higher the infants average WGV. A population‐based Brazilian study involving children under 5 years of age found no association between the prevalence of child overweight and maternal education (Géa‐Horta et al. 2016). Higher maternal education has been shown to contribute positively to better meal and food choices of children, as these women are able to assimilate messages from nutritional education programs and understand the importance of food for promoting health, factors that reduce the risk of overweight in this age group (Schuch et al. 2013). On the other hand, higher maternal education contributes to the absence of mothers from home, with children spending more time watching TV, playing video games, and using computers and thus being exposed to a more sedentary lifestyle, which can culminate in weight gain (Mihrshahi et al. 2011). Also based on some studies (Wehby and López‐Camelo 2017; Shrestha 2020; Laksono et al. 2022; Okhovat et al. 2022), we know that women with higher education levels have greater awareness of healthcare practices and hygiene, which may have contributed to the faster growth rate of their children.

Male infants had higher mean WGV, regardless of the type of delivery. Similarly, Mihrshahi et al. (2011), studying 612 Australian children, showed that male sex is a non‐modifiable factor for rapid weight gain. The difference between sexes can be attributed to anatomical and physiological differences, with boys having larger body structures and more muscle tissue and girls tending to ingest less milk during breastfeeding (Otaigbe et al. 2008; Hoffmann et al. 2021).

Birth weight showed an inverse association with WGV in the first year of life. This finding disagrees with the study by Hoffmann et al. (2021) involving 1783 mother–child pairs, in which birth weight was positively associated with weight in the first year of life. In contrast, two other studies reported that birth weight was not linked to weight in the first year of life (Betoko et al. 2013; Betoko et al. 2017). However, birth weight and WGV in the first year of life must be considered within the context of intrauterine growth. There is evidence that intrauterine growth restriction, which results in low birth weight followed by rapid weight gain after birth, is associated with chronic diseases later in life (Rondó et al. 2013a, 2013b; Lemos et al. 2010; Pereira et al. 2010; Rondó et al. 2008; Pereira‐Freire et al. 2015). To evaluate the effects of the type of delivery on WGV and LGV, we excluded preterm infants from the analysis. However, there were 180 infants with birth weight less than 3000 g, corresponding to 25% of our total population at the end of the one‐year follow‐up period. This fact might explain the inverse association between birth weight and weight at 1 year of age observed in our population. Ong et al. (2000) observed in a sample from the Avon Longitudinal Study involving well‐nourished infants that those with lower birth weight and length, according to the UK growth reference curve standards, exhibited a higher chance of rapid weight gain (RWG) compared to infants positioned at higher points on the curve. Louro et al. (2022) assessed the prevalence of RWG in children born with normal birth weight in Brazil, Peru, Colombia, and Bolivia and its association with overweight at 5 years of age. The authors concluded that RWG in children with normal birth weight was associated with a higher chance of the child becoming obese, with odds ranging from 4.4 in Bolivia to 12.2 in Peru. Woo Baidal et al. (2016) conducted a systematic review to evaluate risk factors for childhood obesity during the first 1000 days of life. According to the review's findings, accelerated infant weight gain was, among others, one of the factors associated with obesity.

In this study, formula‐fed infants showed higher WGV in the first year of life. Similarly, studies have shown that the use of formula is an important predictor of overweight and obesity in children and that breastfed infants have less fat mass than formula‐fed infants (Donovan et al. 2017). The difference in growth between infants fed breast milk and infants fed formula is one of the reasons why the WHO began collecting data to build the childhood growth standards. The higher protein intake that occurs in formula‐fed infants could explain the subsequent weight and length gain when compared to breastfed infants, which has been indicated as a risk factor for future obesity (Donovan et al. 2017).

Infants that exhibited diarrhea episodes in the first year of life had lower WGV. The most important physiological effects of diarrhea are dehydration and malnutrition, which compromise weight gain, thus explaining the influence of this variable on child growth. Studies involving children from the Ecuadorian Amazon demonstrate that trade‐offs in energy allocation, resulting from increased infections and physical activity compared to children in industrialized populations, may interfere with the growth patterns of these children (Urlacher et al. 2019). McDade et al. (2005) investigated C‐reactive protein (CRP) concentrations among children and adolescents from a relatively isolated population in Bolivia and observed that 12.9% exhibited concentrations above 5 mg/L, which is suggestive of inflammation/infection. CRP is an indicator of immune system activation and thus reveals an important pathway through which environmental factors may shape child growth and health.

Maternal characteristics can also influence LGV. In our population, maternal height was associated with LGV, which expresses the influence of maternal factors on infant growth, due to a combination of genetic and non‐genetic factors. Genes influencing skeletal growth, bone development, and overall body size can contribute to longer fetus/infant length (Lunde et al. 2007; Silventoinen et al. 2003; Zhang et al. 2015). Non‐genetic factors include intergenerational influences on growth, as maternal height reflects the cumulative effects of intergenerational nutrition and health, which can influence fetal/infant length (Addo et al. 2013, Ozaltin et al. 2010, Abdulahi et al. 2017).

Similar to the results observed for birth weight and WGV, the relationship between birth length and LGV was also inverse. The shorter the length at birth, the greater the growth in the first year of life. This relationship can be explained by the principles of catch‐up growth (Adair et al. 2013; Godoy et al. 2010), genetic potential, and environmental influences. Infants born lighter or shorter often exhibit faster growth velocities to reach their genetic potential, while those born heavier or longer may show slower growth velocities as they stabilize to a typical growth trajectory. This phenomenon underscores the importance of individualized growth monitoring and support during early childhood.

Diet also influenced length gain in the first year of life. Formula‐fed infants are known to gain more weight and length and to have a higher BMI in the first year of life than infants fed breast milk (Hoffmann et al. 2021). The higher LGV of non‐breastfed infants in the first year of life compared to breastfed infants was therefore expected. The role of breastfeeding as a protective factor against obesity, particularly through its regulation of weight and length accretion, is well known (Carling et al. 2015).

However, it is important to discuss statistical significance and biological significance of the results of this study, considering that they address different aspects of data interpretation and have distinct implications. Moreover, it is well known that differences among populations further complicate the interpretation of the results, as genetic, environmental, and behavioral factors can influence research outcomes.

Statistical significance depends on sample size, variability, and effect size, and does not indicate the magnitude or practical importance of the effect. On the other hand, biological significance often considers effect size and clinical relevance (Rothwell 2005; Valeggia and Fernández‐Duque 2021). Birth weight, for example, although statistically significant, did not show an important effect size on WGV, probably because in this population most children were born with an appropriate weight for their gestational age. The same observation also applies to maternal height in relation to the LGV.

The prospective cohort design was a strength of this study, since the results are generally more reliable. Moreover, a GEE is a more appropriate statistical tool to evaluate the effect of time on the outcomes investigated, since it can accommodate missing data, which are relatively common in cohort studies (Guimarães Santos Pinto and Naomi Hirakata 2012; Liang and Zeger 1986). However, the main limitations of our study were the losses to follow‐up due to the COVID‐19 pandemic, lack of assessment of maternal mental health, which according to some studies (Rondó et al. 2013a, 2013b; Oyetunji and Chandra 2020), may also influence infant growth, and the difficulty in differentiating between elective and planned cesarean sections.

## 5 Conclusion

The factors that explain WGV and LGV in the present population are more closely related to maternal characteristics, such as height and educational attainment, birth characteristics, feeding behavior, and morbidity than to the type of delivery. Investigating growth trajectories from birth to early childhood is essential because they are associated with the risk of chronic diseases later in life.

## Author Contributions

Roseanne de Sousa Nobre and Patrícia Helen de Carvalho Rondó: conceptualization and methodology. Roseanne de Sousa Nobre, Paula Louro Silva, Letícia Falcão de Carvalho, Jéssica Lana Sales Lacerda, Lívia Patrícia Rodrigues Batista, Tamiris Ramos‐Silva, Natália Pinheiro‐Castro, Liania Alves Luzia: investigation. Roseanne de Sousa Nobre, Tamiris Ramos‐Silva, Natália Pinheiro‐Castro: statistical analysis, writing‐original draft. Patrícia Helen de Carvalho Rondó: funding acquisition, supervision, project administration, critically reviewing the manuscript. All authors: writing‐review and editing.

## Ethics Statement

The study was approved by the Ethics Committee of the School of Public Health, University of São Paulo (No. 59787216.2.0000.5421) and followed the guidelines established by the Declaration of Helsinki.

## Conflicts of Interest

The authors declare no conflicts of interest.

## Data Availability

The data that support the findings of this study are available from the corresponding author upon reasonable request.
